# Excitation of skeletal muscle is a self-limiting process, due to run-down of Na^+^, K^+^ gradients, recoverable by stimulation of the Na^+^, K^+^ pumps

**DOI:** 10.14814/phy2.12373

**Published:** 2015-04-12

**Authors:** Torben Clausen

**Affiliations:** Department of Biomedicine, Aarhus UniversityAarhus C, Denmark

**Keywords:** K^+^-fatigue, Na^+^, skeletal muscle

## Abstract

The general working hypothesis of this study was that muscle fatigue and force recovery depend on passive and active fluxes of Na^+^ and K^+^. This is tested by examining the time-course of excitation-induced fluxes of Na^+^ and K^+^ during 5–300 sec of 10–60 Hz continuous electrical stimulation in rat extensor digitorum longus (EDL) muscles in vitro and in vivo using ^22^Na and flame photometric determination of Na^+^ and K^+^. 60 sec of 60 Hz stimulation rapidly increases ^22^Na influx, during the initial phase (0–15 sec) by 0.53 *μ*mol(sec)^−1^(g wet wt.)^−1^, sixfold faster than in the later phase (15–60 sec). These values agree with flame photometric measurements of Na^+^ content. The progressive reduction in the rate of excitation-induced Na^+^ uptake is likely to reflect gradual loss of excitability due to accumulation of K^+^ in the extracellular space and t-tubules leading to depolarization. This is in keeping with the concomitant progressive loss of contractile force previously demonstrated. During electrical stimulation rat muscles rapidly reach high rates of active Na^+^, K^+^-transport (in EDL muscles a sevenfold increase and in soleus muscles a 22-fold increase), allowing efficient and selective compensation for the large excitation-induced passive Na^+^, K^+^-fluxes demonstrated over the latest decades. The excitation-induced changes in passive fluxes of Na^+^ and K^+^ are both clearly larger than previously observed. The excitation-induced reduction in [Na^+^]_o_ contributes considerably to the inhibitory effect of elevated [K^+^]_o_. In conclusion, excitation-induced passive and active Na^+^ and K^+^ fluxes are important causes of muscle fatigue and force recovery, respectively.

## Introduction

In skeletal muscle, excitation and action potentials are elicited by passive fluxes of Na^+^ and K^+^ across the sarcolemma and t-tubular membranes. These fluxes have been quantified in several intact muscle preparations (Creese et al. 1958; Hodgkin and Horowicz 1959; Clausen and Kohn 1977; Juel 1986; Nagaoka et al. 1994; Clausen 2003; Clausen et al. 2004) and it is generally assumed that they account for the charge movements required to trigger the action potentials and contractions. The inevitable cost of the action potentials is a rapid rise in extracellular K^+^ ([K^+^]_o_), and decrease in [Na^+^]_o_ and already long ago it was proposed that the K^+^ lost from working muscles was “one of the factors which causes the intensity of contraction to decrease” (Fenn 1940). Measurements of the intracellular concentration of K^+^ in biopsies of human vastus lateralis muscle showed that intense dynamic knee extension caused a reduction from 165 to 129 mmol L^−1^ (Sjøgaard et al. 1985). This corresponds to a decrease of 36 mmol L^−1^, sufficient to cause a substantial increase in the extracellular K^+^ of the working muscle. However, in vivo measurements of interstitial K^+^ with microdialysis probes showed that in human working muscles, extracellular K^+^ increased by only 9 mmol L^−1^ (Juel et al. 2000), 11 mmol L^−1^ (Green et al. 2000), or 12 mmol L^−1^ (Nordsborg et al. 2003), suggesting that excitation-induced rise in the interstitial concentration of K^+^ might only constitute a modest contribution to muscle fatigue. On the other hand, recent studies indicate that the excitation-induced passive fluxes of Na^+^ and K^+^ in working rat skeletal muscles is sufficient to augment the extracellular concentration of K^+^ to values around 50 mmol L^−1^ both in vitro and in vivo, accompanied by a comparable decrease in [Na^+^]_o_ (Clausen 2008a, 2011, 2013). Such high values were unexpected, but sufficient to cause fatigue.

Due to these discrepancies, this study was initiated to obtain a closer analysis of the time-course of changes in the excitation-induced loss of K^+^ and force decline in rat soleus and extensor digitorum longus (EDL) muscles. In EDL muscles passive Na^+^, K^+^-fluxes, [Na^+^]_i_, [Na^+^]_o_ and [K^+^]_o_ were supplemented by ^22^Na-fluxes. There is good evidence that in skeletal muscle, the excitation-induced rate of Na^+^-influx is similar to K^+^-efflux (Hodgkin and Horowicz 1959; Balog and Fitts 1996) and in some studies they are almost identical (Everts and Clausen 1988; Clausen et al. 2004).

It has been observed that in rat EDL muscle stimulated at 60 Hz for 30 sec, the excitation-induced loss of K^+^ is around fivefold larger in the first 15 sec of excitation than in the last 15 sec (Fig.1 in Clausen 2011). This was attributed to progressive loss of excitability induced by the gradual rise in [K^+^]_o_. Striking evidence for a role of K^+^ in muscle fatigue also emerged from the observation that in rat EDL muscle, the rate of force decline during continuous stimulation at 60 Hz, is 5.9-fold faster than in soleus (Clausen et al. 2004). The same study showed that in EDL, the efflux of K^+^ per action potential is 6.6-fold larger than in soleus, in keeping with the relative difference in force decline of the two muscles (Clausen et al. 2004). The mechanism can be related to the observation that excitation-induced ^22^Na-influx in EDL is 90% higher than in soleus, possibly because EDL contains 70% more Na^+^ channels per g wet wt. than soleus (Gissel and Clausen 2000).

**Figure 1:**
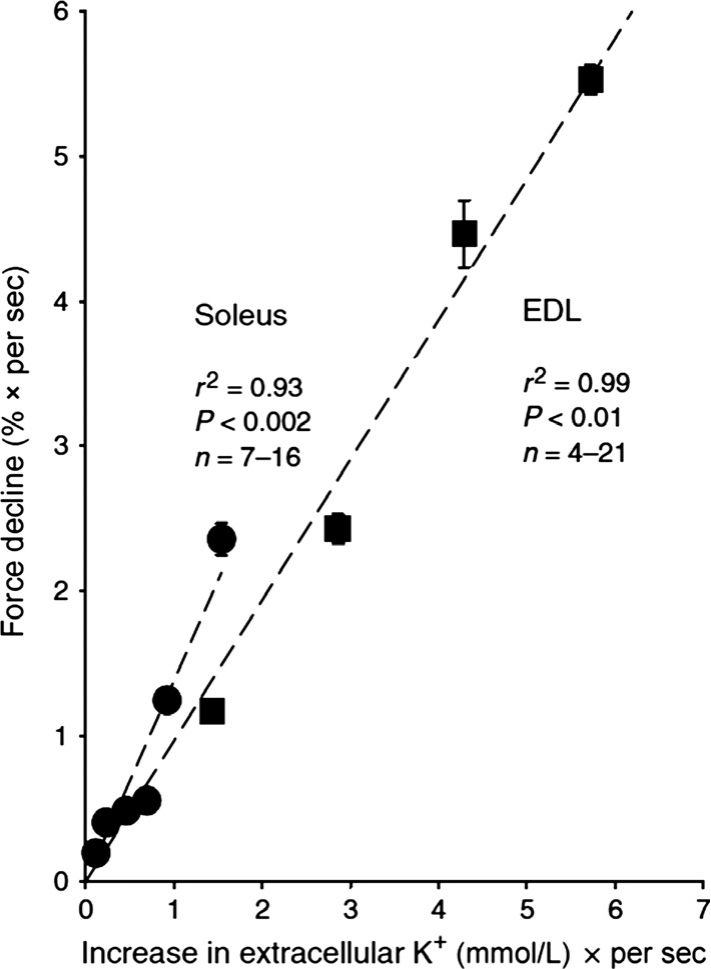
Relationship between the rate of excitation-induced increase in [K^+^]_o_ and the rate of force decline in isolated rat muscles. Soleus and extensor EDL muscles were prepared from 4-week-old rats, mounted for isometric contractions in force transducers and incubated in KR buffer at 30°C. The muscles were exposed to direct continuous stimulation at frequencies 20–200 Hz (1 ms pulses at 10 V). The rate of force decline was recorded and expressed as per cent per second of the maximum force recorded within 0.5–1.5 sec after the onset of stimulation. Each point represents the mean ± SEM of observations on 4–21muscles. Reproduced, with permission from Fig.4 in Clausen 2008b). For details, see the same reference.

As described in a published FEPS honorary lecture (Clausen 2008b), both in isolated rat soleus and EDL muscles, the initial rates of force decline induced by continuous electrical stimulation at varying frequencies (10–200 Hz) were found to be closely correlated with the rates of increase (in mmol L^−1^ (sec)^−1^) in [K^+^]_o_ (*r*^2^ = 0.93, *P* < 0.002 for soleus, and for EDL muscles (*r*^2^ = 0.99, *P* < 0.01), respectively (see Fig.1). The correlation curves for the two muscles almost fall in line, indicating that in both muscles, the endurance depends on the rate of excitation-induced increase in interstitial K^+^. These observations confirm in more detail those already obtained by Nagaoka et al. (1994) by comparing contracting rat EDL and soleus muscles.

Muscle fatigue is often proposed to be a multifaceted process caused by several different cellular mechanisms (see fig. 8 in Allen et al. 2008). However, the relative contribution of the proposed mechanisms has rarely been quantified or directly compared. This study focus on the early time-course (0–60 sec) of excitation-induced gain of Na^+^ and loss of K^+^ as measured by flame photometry during continuous stimulation at 60 Hz. In order to obtain alternative information with an isotopic tracer for Na^+^, these observations were supplemented by measurements of ^22^Na influx and ^22^Na content, allowing better recording of the first 5 sec of stimulation, the later changes in the rate of Na^+^ uptake and [Na^+^]_o_.

The passive leaks of Na^+^ and K^+^ were compared with the capacity for restorative Na^+^, K^+^-pump-mediated efflux of Na^+^ and influx of K^+^. Possible mechanisms of excitation and fatigue were explored by rather detailed bookkeeping of Na^+^, K^+^ exchange and Na^+^, K^+^-contents in the various compartments of the intact working muscle. It has been shown that a decrease in [Na^+^]_o_ in the incubation medium leads to reduction in contractile force (Bouclin et al. 1995; Overgaard et al. 1997; Cairns et al. 2003; Cairns and Lindinger 2008). Excitation-induced influx of Na^+^ is associated with an equimolar efflux of K^+^ and an increase in [K^+^]_o_, which is often sufficient to be a likely cause of muscle fatigue (Clausen 2003). Moreover, as a decrease in [Na^+^]_o_ clearly augments the inhibitory effect of the increased [K^+^]_o_ on excitability and contractility it is important to quantify these rather large changes.

As the gating of Na^+^ channels has a temperature coefficient (Q_10_) of around 3 (Hille 2001), the effect of increasing the incubation temperature from 30 to 37°C on excitation-induced ^22^Na uptake was examined. This was further justified by the fact that the body temperature of rats is around 38°C (Felies et al. 2004) and at this more physiological temperature, active Na^+^, K^+^-transport in rat muscle is augmented, showing a temperature coefficient (Q_10_) of 2.3 (Clausen and Kohn 1977).

## Materials and Methods

### Animals and ethical approval

All handling and use of rats complied with Danish animal welfare regulations, including the euthanasia, which in addition was approved by the Animal Welfare Officer of Aarhus University. Animals were killed by cervical dislocation, followed by decapitation. All experiments (on muscles from a total of 80 animals) were performed using 4–6-week-old female and male Wistar rats (no GM animals) bred at the Department of Biomedicine, Aarhus University. The animals weighed 60–80 g, were fed ad libitum and kept in a thermo-stated environment at 21°C with a 12/12 h light/dark cycle.

### In vitro experiments

Most experiments were performed in vitro at 30°C using Krebs-Ringer bicarbonate buffer (KR) containing the following (in mmol L^−1^): 122.2 NaCl, 25 NaHCO_3_, 2.8 KCl, 1.2 KH_2_PO_4_, 1.2 MgSO_4_, 1.3 CaCl_2_, and 5.0 D-glucose. The pH was maintained at 7.4 by continuous gassing with a mixture of 95% O_2_ and 5% CO_2_, humidified at 30°C. Intact soleus or extensor digitorum longus (EDL) muscles weighing 20–30 mg were dissected out during wash with a 154 mmol L^−1^ NaCl solution at room temperature, mounted for isometric contractions at resting length in holders, surrounded with platinum wire electrodes and equilibrated at 30°C for 15 min in KR containing ^22^Na (1 *μ*Ci (mL)^−1^. To assess the possible role of this low temperature, some experiments were performed at 37°C. During the last 5, 15, or 60 sec of the incubation with ^22^Na, the muscles were stimulated via the platinum electrodes using 0.2 ms 10 V pulses. Immediately after these stimulation periods, the extracellular Na^+^, K^+^, and ^22^Na were removed by washing the muscles four times 15 min in an ice-cold Na^+^-free Tris-sucrose buffer containing the following (in mmol L^−1^): 263 sucrose, 10 Tris-HCl, 2.8 KCl, 1.3 CaCl_2_, 1.2 MgSO_4_, and 1.2 KH_2_PO_4_, pH 7.4. This buffer was gassed with air, humidified at 0°C. We have previously shown that during wash in this buffer at 0°C, the K^+^ content of resting EDL muscles remains constant from 0 to 150 min (Everts and Clausen 1992), whereas intracellular Na^+^ content decreases at a constant rate from 40 to 150 min. This slow decrease in Na^+^ content can be corrected for (adjusted back to the start of washout at 0°C) by multiplying with a constant of 1.59 per hour of washout (Murphy et al. 2006). The K^+^ content of resting EDL muscles showed no significant change during washout in the ice-cold Na^+^-free buffer for 60 or 120 min (Clausen 2013). For the quantification and interpretation of the excitation-induced loss of K^+^ and gain of Na^+^ at 30°C, it is important that during the subsequent washout at 0°C, there is no loss of cellular K^+^, neither in resting nor in muscles stimulated in KR at 30°C prior to the washout (Everts and Clausen 1992). Moreover, as the washout at 0°C is performed in a Na^+^-free buffer, the excitation-induced increase in Na^+^ content cannot take place during this washout, but must have happened during the prior stimulation at 30°C in KR. Control experiments showed that when the washout time was reduced from 60 to 20 min, there was no reduction in the net loss of intracellular K^+^, also indicating that this loss does not take place during the washout at 0°C (Clausen 2013).

As can be seen in Table1, neither resting nor stimulated (60 Hz for 60 sec) EDL muscles show any decrease in total K^+^ content during a subsequent 20–60 min of washout in ice-cold Tris-sucrose buffer. In an earlier study (Clausen 2011) possible loss of cellular integrity due to electroporation was tested by measuring the content of the intracellular enzyme LDH in the ice-cold Tris-sucrose washout buffer after EDL muscles had been incubated at 30°C in KR for 60 sec during 60 Hz stimulation. This showed no LDH in the Tris-sucrose buffer (Clausen 2011). In a separate project performed by Dr. Hanne Gissel, dry weight and total water content as determined by drying EDL muscles overnight at 60°C to constant weight were 24.2 and 75.8 per cent, respectively (*N* = 33 muscles from in vitro experiments). Intracellular water space was calculated by deducting the ^14^C-sucrose space measured in EDL muscles in vitro (Clausen et al. 2004) from total water content (0.758 mL (g wet wt.)^−1^ – 0.214 mL (g wet wt.)^−1^ = 0.544 mL (g wet wt.)^−1^.

**Table 1:** Total K^+^ contents in rat EDL muscles. Effects of incubation in KR buffer at 30 °C for 60 sec without or with stimulation at 60 Hz followed by wash for 20–60 min in ice-cold Na^+^-free Tris-sucrose buffer, blotting, weighing and flame photometric determination of K^+^ content. Mean values of measurements on groups of five muscles are given as *μ*moles (g wet wt.)^−1^ ±  SEM.

Duration of washout	Resting muscles	Stimulated muscles
20 min	102.3 ± 2.1	95.8 ± 1.6
40 min	103.9 ± 0.8	93.6 ± 3.6
60 min	97.9 ± 2.8	93.3 ± 1.3

### In vivo experiments

To assess the possible role of preserved circulation, stimulation experiments were also performed in vivo using a total 22 rats, each anesthetized by an intraperitoneal injection of 0.11 mL of a solution containing 0.55 mg fluanisone, 0.0173 mg fentanyl citrate, and 0.275 mg midazolam, causing full analgesia in 10–15 min. These experiments were performed without ^22^Na solely exploring the time-course of Na^+^, K^+^-exchange during 60 Hz of stimulation for 5–60 s. When analgesia could be ascertained, the skin covering the frontal surface of the hind leg was opened, and the tendon of the tibialis anterior muscle grasped using a surgical forceps. The muscle was gently and slowly drawn aside so that the EDL muscle could be reached. The two branches of a platinum wire electrode were placed around the mid-portion of the EDL and the muscle was stimulated from 5 to 60 sec at its resting length using 0.2 ms 10 V pulses at 60 Hz. Immediately after the cessation of stimulation, ice-cold Na^+^-free Tris-sucrose buffer was dripped from a pipette on the muscle to obtain rapid cooling and inhibition of the efflux of K^+^ and Na^+^, K^+^-pump-mediated reaccumulation of K^+^. 5 sec later, the muscle was cut free across the tendon, carefully avoiding damage to the muscle fibers. Then the muscle was transferred to a perforated polyethylene cylinder and like in the in vitro experiments washed four times 15 min in ice-cold Na^+^-free Tris-sucrose buffer during continuous gassing with air, blotted on dry filter paper and taken for TCA extraction and flame photometric determination of Na^+^ and K^+^. Using ^14^C-sucrose, the extracellular water space measured in vivo (without washout in ice-cold Tris-sucrose) was found to amount to 0.177 mL (g wet wt.)^−1^ ± 0.010 in EDL muscles stimulated for 60 sec at 60 Hz (*N* = 20). In resting EDL muscles, ^14^C-sucrose space measured in vivo was 0.182 ± 0.011 mL (g wet wt.)^−1^ (*N* = 19). For detailed description of these in vivo experiments, see Clausen 2013).

### Chemicals and isotopes

All chemicals used were of analytical grade. Ouabain was from Sigma. ^14^C-sucrose (435 mCi/mmol) and ^22^Na 17.6 Ci/mmol were obtained from GE Healthcare.

### Statistics

All data are presented as means ± SEM. The statistical significance of a difference between two groups was ascertained with the Student's two-tailed *t*-test for nonpaired observations using the program Sigma Plot 12.

## Results

**Figure 2:**
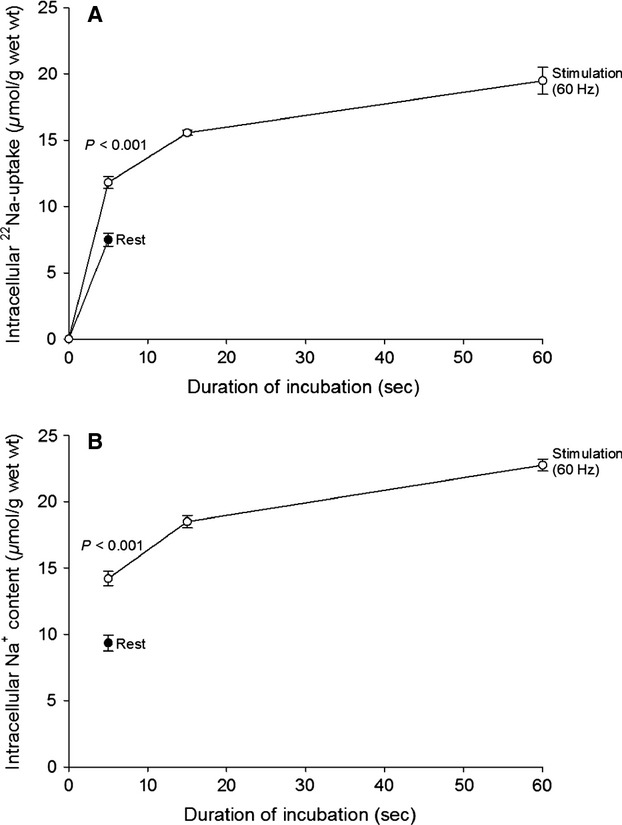
Time-course of changes in intracellular ^22^Na-uptake (A) and Na^+^-contents (B) in isolated rat EDL muscle in vitro during stimulation at 60 Hz for 5–60 s. Intact EDL muscles were mounted for isometric contractions in holders, surrounded with platinum wire electrodes and equilibrated at 30°C for 15 min in KR containing ^22^Na (1 *μ*Ci (mL)^−1^. During the last 5, 15 or 60 sec of this incubation, the muscles were stimulated via the electrodes using 0.2 ms 10 V pulses given at 60 Hz 300, 900, and 3,600 times, respectively. To remove ^22^Na, Na^+^ and K^+^ from the extracellular space, all muscles were then washed 4 × 15 min in ice-cold Na^+^-free Tris-sucrose buffer, blotted, weighed and taken for counting of ^22^Na and flame photometric determination of Na^+^ and K^+^ contents. The contents of ^22^Na and Na^+^ were corrected for loss from the intracellular pools during the 4 × 15 min washout in the cold. A previously determined correction factor of 1.59 per hour of washout was used (Murphy et al. 2006). Each point is the mean of observations ± SEM on six resting vs. six contralateral stimulated muscles.

From Figure2A, it can be seen that during continuous stimulation for 60 sec at 60 Hz, the intracellular uptake of ^22^Na shows an early rapid increase, followed by a much slower phase. When compared with the resting muscles, the uptake of ^22^Na in the stimulated muscles is significantly larger already within the first 5 sec of stimulation (*P* < 0.001). During the first 15 sec of stimulation, the total uptake of ^22^Na amounts to 15.5 *μ*mol/g wet wt., and when corrected for the resting uptake of 7.5 *μ*mol/g wet wt., the stimulation-induced net uptake of ^22^Na amounts to 8 *μ*mol/g wet wt. or 0.53 *μ*mol/g wet wt.(sec)^−1^. During the last 45 s, (from 15 to 60 s) there is a further ^22^Na uptake of only 4.0 *μ*mol/g wet wt., corresponding to 0.089 *μ*mol/g wet wt.(sec)^−1^ (Fig.2A). Thus, the initial rate (per second) of ^22^Na uptake (from 0 to 15 sec) is sixfold faster (0.53/0.089 = 6.0) than the late (15–60 sec) rate of ^22^Na uptake. At the end of the 60 sec stimulation, the total ^22^Na-uptake had reached 19.5 *μ*mol/g wet wt. When corrected for the resting uptake of 7.5 *μ*mol/g wet wt. this amounts to 12.0 *μ*mol/g wet wt. (Fig.2A). As shown in Figure2B, the intracellular content of Na^+^ as measured by flame photometry reaches 18.5 *μ*mol/g wet wt. during the first 15 sec of 60 Hz stimulation and during the last 45 sec there is a further uptake of 4.3 *μ*mol/g wet wt, corresponding to 0.096 *μ*mol/g wet wt.(sec)^−1^, in good agreement with the ^22^Na-uptake of 0.089 *μ*mol/g wet wt.(sec)^−1^. *N* = six versus six muscles.

**Figure 3:**
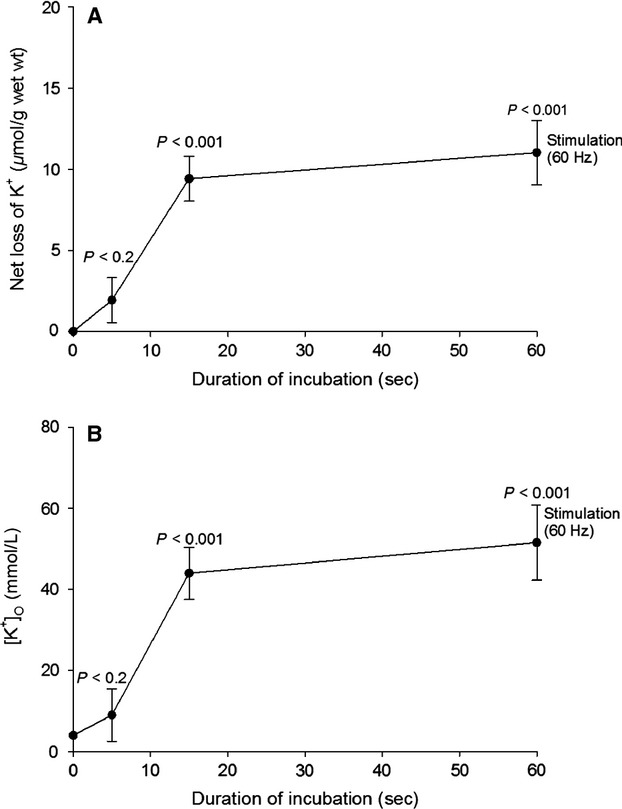
Time-course of net loss of K^+^ (A) and increase in [K^+^]_o_ (B) measured in the experiments shown in Fig.2. The excitation-induced net loss of K^+^ was calculated as the difference between total K^+^ contents of resting muscles and that of stimulated muscles. The statistical significance of the difference between these two sets of values is given by P. The mean increase in [K^+^]_o_ was calculated by dividing the net loss of K^+^ by the ^14^C-sucrose space (0.214 mL (g muscle wet wt.)^−1^, measured in isolated EDL muscles (Clausen 2008a). Each point is the mean ± SEM of observations on six resting vs. six contralateral stimulated muscles. The starting level of [K^+^]_o_ was assumed to be 4 mmol L^−1^ like in the standard K.R., as indicated at 0 sec.

The uptake of ^22^Na and net Na^+^ uptake are summarized in Table2, which shows the effects of continuous electrical stimulation of rat EDL muscles using 0.2 ms 10 V pulses at 60 Hz for 5, 15, or 60 sec. When expressed in nmol/pulse, the uptake rates of ^22^Na and Na^+^ were both about 4.4-fold (14.4/3.3 = 4.436 and 16.2/3.7 = 4.378) larger in the first 5 sec than in the entire stimulation period of 60 sec, reflecting the progressive loss of excitability due to accumulation of K^+^ in the extracellular space (Clausen 2011). To detect this K^+^ accumulation in the ^22^Na-influx experiment shown in Figure2, the time-course of concomitant changes in excitation-induced net loss of K^+^ and extracellular concentrations of K^+^ ([K^+^]_o_) were followed and illustrated in Figure3A and B, respectively.

**Table 2:** Stimulation induced ^22^Na uptake and net increase in intracellular Na^+^ content in rat EDL muscles in vitro. All values for Na^+^ represent the increase over and above the ^22^Na uptake or Na^+^ content in resting muscles. The uptake per stimulation pulse is calculated by dividing the Na^+^ values by the number of pulses (300–3600) given in the experiment described in legend to Fig.2. Each value is the mean of observations on six resting or stimulated muscles ± SEM.

60 Hz stim.	^22^Na uptake	Net increase in i.c. Na^+^	^22^Na^+^ uptake	Net Na^+^ uptake
	*μ*mol (g wet wt.)^−1^	*μ*mol (g wet wt.)^−1^	nmol (pulse)^−1^	nmol (pulse)^−1^
5 sec	4.3 ± 0.3	4.9 ± 0.8	14.4 ± 1.0	16.2 ± 2.7
15 sec	8.0 ± 0.2	9.2 ± 0.8	9.0 ± 0.3	10.2 ± 1.3
60 sec	12.0 ± 0.5	13.4 ± 0.7	3.3 ± 0.1	3.7 ± 0.2

[K^+^]_o_ was calculated by dividing the excitation-induced net loss of K^+^ by the ^14^C-sucrose space measured in EDL muscles in vitro (0.214 mL/g wet wt., Clausen et al. 2004). After 15 and 60 sec of stimulation [K^+^]_o_ increases by 44 mmol L^−1^ (9.4 *μ*mol/0.214 mL; *P* < 0.001) and 51 mmol L^−1^ (10.9/0.214 mL; (*P* < 0.001), respectively, sufficient to induce pronounced inhibition of contractile force. (see Cairns et al. 2003 and Fig.3 in Nielsen and de Paoli 2007). Thus, previous experiments with rat EDL performed under the same conditions (isometric contractions in KR buffer at 30°C with 0.2 ms 10–12 V pulses at 60 Hz) showed that after 15 and 60 s, force had decreased by 30 and 93%, respectively, as compared with initial force (Clausen 2011). Earlier experiments with rat EDL showed a 50% loss of force after 15 sec and around 90% loss after 30 sec of 60 Hz stimulation, respectively (Clausen et al. 2004).

The mechanisms of the clearance of K^+^ from the extracellular space was previously (Clausen 2008a) examined by comparing the loss of K^+^ during stimulation in KR buffer with muscles where the buffer had been removed, preventing the clearance by diffusion into surrounding buffer. After 30 sec of 60 Hz stimulation, the average concentration of K^+^ in the interstitial water space reached the same level (48.6 mmol L^−1^) as in the muscles stimulated in air (46.7 mmol L^−1^) (*N* = 9 vs. 7, *P* = 0.87). This indicates that K^+^ is not primarily cleared by diffusion, but by reaccumulation via the Na^+^,K^+^ pumps into the cells. In keeping with this, the omission of KR buffer caused no interference with contractile performance, when compared with muscles kept in KR buffer neither in EDL, nor in soleus muscle (Clausen 2008a). In contrast, ouabain-induced inhibition of K^+^ clearance via the Na^+^,K^+^ pumps markedly reduced contractile endurance and force recovery in air (Clausen 2008a). In keeping with this, ouabain as well as downregulation of the muscle contents of Na^+^,K^+^ pumps induced by prior K^+^ depletion of the rats were found to induce a graded reduction of the contractile endurance in rat soleus (Nielsen and Clausen 1996).

**Figure 4:**
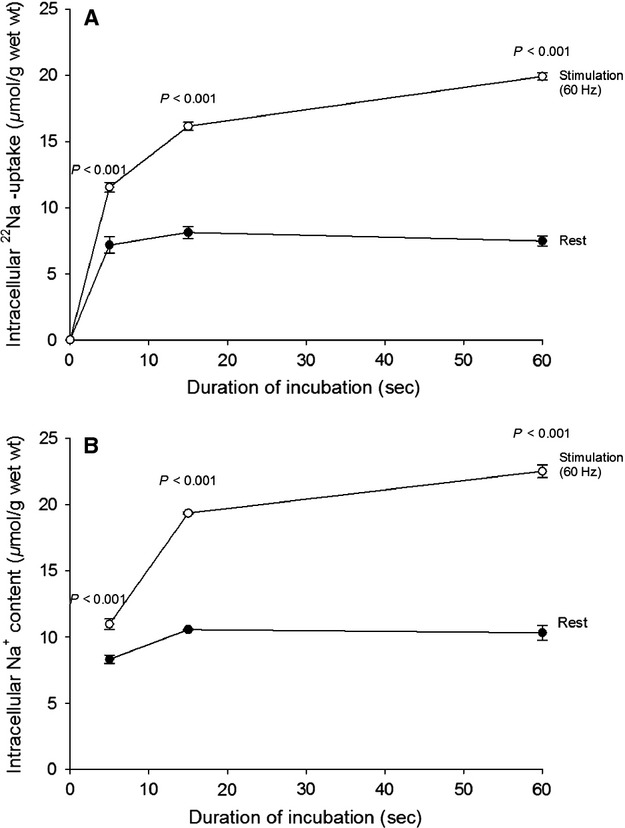
Time-course of changes in intracellular ^22^Na uptake (A) and Na^+^ content (B) in rat EDL muscle in vitro during rest or stimulation at 60 Hz for 5–60 sec. Experimental conditions and calculations as described in the legend to Fig.2, except that during the 15 min of exposure to ^22^Na terminated by 5–60 sec stimulation, muscles were mounted in KR buffer at 37°C. This incubation was also followed by 4 × 15 min of washout in ice-cold Na^+^-free Tris-sucrose buffer, blotting, weighing, counting of ^22^Na and flame photometric measurements of Na^+^ and K^+^ contents. Each point is the mean of observations on three to six muscles ± SEM with *P*-values indicating the statistical significance of the differences between the resting and the stimulated muscles.

The effects of increasing the incubation temperature from 30 to 37°C on excitation-induced ^22^Na uptake was examined. As shown in Figure4A and B as compared with Figure2A and B, respectively, this increase in temperature caused no significant change in the effect of 60 Hz stimulation on ^22^Na uptake or Na^+^ content. It should be noted that during the 60 sec of incubation at 37°C, ^22^Na uptake and Na^+^ content show no significant change in the resting muscles. *N* = 3 versus six muscles.

**Figure 5:**
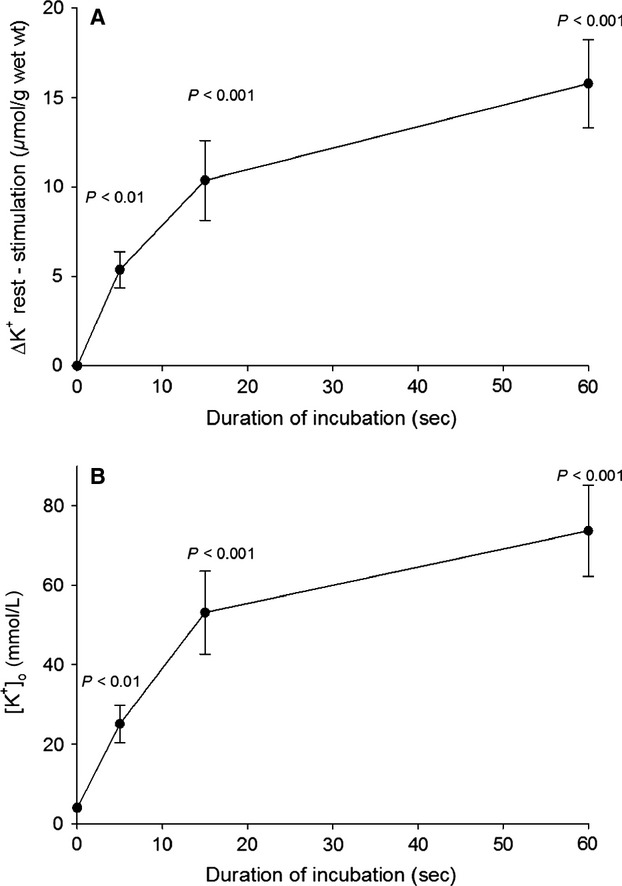
Time-course of net loss of K^+^ (A) and increase in [K^+^]_o_ (B) measured in the experiments shown in Fig.4 at 37°C. Calculations as described in the legend to Fig.2. The starting level of [K^+^]_o_ was assumed to be 4 mmol L^−1^ like in the KR buffer, indicated at 0 sec. Each point is the mean of observations on four to six muscles ± SEM with *P*-values indicating the statistical significance of the differences between the resting and the stimulated muscles.

As shown in Figure5A and B, at 37°C 60 Hz stimulation causes somewhat larger net loss of K^+^ and increase in [K^+^]_o_ than at 30°C (shown in Fig.3A and B, respectively). As the Q_10_ of the Na^+^, K^+^ pumps is 2.3 (Clausen and Kohn 1977), this could in part be due to the augmented rate of active Na^+^, K^+^ transport at 37 °C and ensuing better conservation of excitability and larger release of K^+^.

As shown in Table3, the effect of electrical stimulation was also tested under more physiological conditions using 1 ms 10 V pulses at 10 Hz in vivo. After 300 sec of continuous stimulation, EDL muscles show a net loss of K^+^ of 12.5 *μ*mol (g wet wt.)^−1^ into the extracellular space of 0.177 mL (g muscle wet wt.)^−1^ (*N* = 4 vs. 4). This implies that [K^+^]_o_ in the stimulated muscles increases by 12.5 *μ*mol (g wet wt.)^−1^/0.177 mL = 70.6 mmol L^−1^. Concomitantly, the intracellular content of Na^+^ increases to 26.2 *μ*mol (g wet wt.)^−1^. When distributed in the intracellular water space of 0.544 mL (g wet wt.)^−1^, this corresponds to an intracellular Na^+^ concentration of 48.2 mmol L^−1^ (26.2 *μ*mol (g wet wt.)^−1^/0.544 mL).

**Table 3:** Effects of continuous 10 Hz stimulation in vivo on K^+^ loss, [K^+^]_o_, and [Na^+^]_i_ in rat EDL muscles. As described in Materials and Methods, four rats were anesthetized and EDL muscles were stimulated at resting length for 300 sec using 10 V pulses of 1 ms duration at a frequency of 10 Hz. The K^+^ content of the stimulated muscles was deducted from that of the resting contralateral muscles and [K^+^]_o_ calculated by dividing that difference in K^+^ content by the ^14^C-sucrose space (0.177 mL/g wet wt. measured in earlier in vivo experiments (Clausen 2013)). Each value is the mean of observations on four muscles ± SEM.

Total net K^+^ loss	12,500 ± 2200 nmol (g wet wt.)^−1^ (*P* < 0.001)
K^+^ loss (s)^−1^	12,500 ± 2200/300 nmol(g wet wt.)^−1^ = 42 ± 7 nmol (g wet wt.)^−1^
K^+^ loss per pulse	42 ± 7/60 nmol = 0.7 ± 0.12 nmol (g wet wt.)^−1^
[K^+^]_o_	12.5 ± 2.2/0.177 *μ*mol = 70.6 ± 12.4 mmol L^−1^
[Na^+^]_i_	48.0 ± 2.4 mmol L^−1^

**Figure 6:**
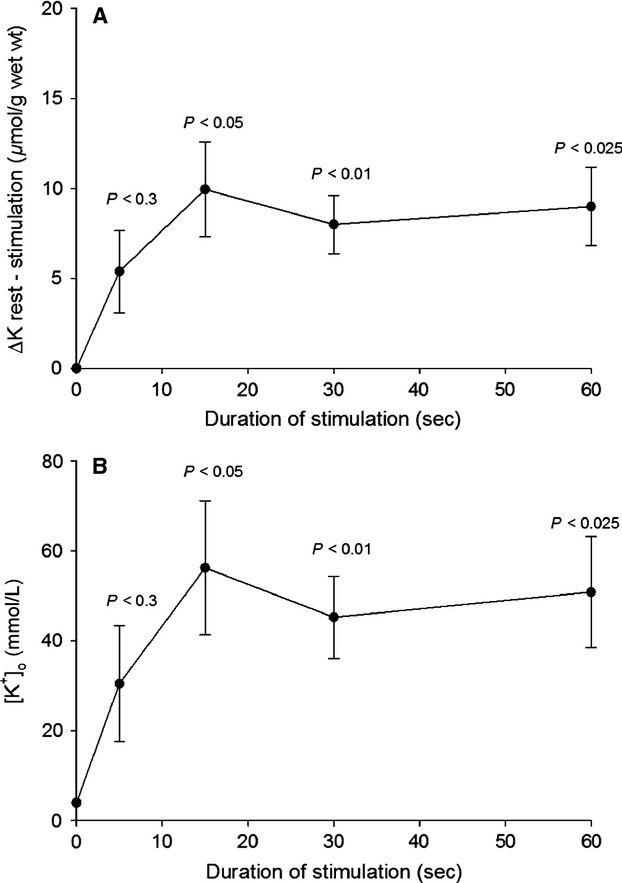
Time-course of the effects of electrical stimulation in vivo on net loss of K^+^ (A) and the increase in [K^+^]_o_ (B). As described in Materials and Methods, rats were anesthetized and the EDL muscles stimulated at resting length using platinum wire electrodes and 0.2 ms 10 V pulses at 60 Hz for 5–60 sec. The increase in [K^+^]_o_ was calculated by dividing the net loss of K^+^ by the ^14^C-sucrose space (0.177 mL (g wet wt.)^−1^ measured in earlier in vivo experiments (Clausen 2013). The starting level of [K^+^]_o_ was assumed to be 4 mmol L^−1^ like in rat plasma, indicated at 0 sec. Each point is the mean of observations on four to seven resting or stimulated muscles ± SEM with *P*-values indicating the statistical significance of the differences between the resting and the stimulated muscles.

Figure6 shows the excitation-induced net loss of K^+^ after 5, 15, 30, and 60 sec of continuous stimulation at 60 Hz (6A) and the increase in [K^+^]_o_ (6B) measured in vivo in rat EDL muscles. After 5 sec of stimulation, the loss of K^+^ was 5.4 *μ*mol (g wet wt.)^−1^, corresponding to an increase in [K^+^]_o_ of 5.4 (g wet wt.)^−1^/0.177 mL = 30.5 mmol L^−1^ (not significant). Somewhat larger losses of K^+^ were recorded after 15, 30, and 60 sec of stimulation, and the differences between K^+^ contents (ΔK) of resting and stimulated EDL muscles were all significant after these durations of stimulation (*P* < 0.01–0.05). For each group of rats, mean [K^+^]_o_ was calculated by dividing the net loss of K^+^ by the ^14^C-sucrose space of 0.177 mL/g muscle wet wt. measured in vivo (Clausen 2013). In vivo, electrical stimulation caused slightly lower loss of cellular K^+^, but slightly higher increase in [K^+^]_o_ than in vitro.

## Discussion

The novelty of the present results is that the excitation-induced increase in [K^+^]_o_ is considerably larger than previously observed using microdialysis probes (Green et al. 2000; Juel et al. 2000; Nordsborg et al. 2003). As the extracellular water space in rat EDL muscle is rather narrow (0.214 mL (g wet wt.)^−1^ compared to the intracellular water space (0.544 mL (g wet wt.)^−1^, the excitation-induced changes of the extracellular concentrations of Na^+^ and K^+^ are relatively large. More importantly, the excitation-induced drop in [Na^+^]_o_ may add considerably to the fatigue caused by the increase in [K^+^]_o_ in the extracellular space and possibly also in the t-tubular lumen (Adrian and Peachey 1973; Bouclin et al. 1995; Overgaard et al. 1997; Cairns et al. 2003; Cairns and Lindinger 2008). In the isolated rat EDL muscle, the extracellular space as measured using ^14^C-sucrose amounts to 0.214 mL (g muscle wet wt.)^−1^ (Clausen et al. 2004). With an extracellular Na^+^ concentration of 147 mmol L^−1^, this corresponds to an extracellular pool of Na^+^ of 31.5 *μ*mol (g muscle wet wt.)^−1^ (0.214 mL × 147 *μ*mol (mL)^−1^. As mentioned above, the present results show that during continuous stimulation for 60 sec at 60 Hz, the excitation-induced intracellular uptake of ^22^Na amounts to 12.0 *μ*moles (g wet wt.)^−1^ (Fig.2A), and the net increase in intracellular Na^+^ content measured by flame photometry is 13.4 *μ*moles (g wet wt)^−1^ (Fig.2B). This corresponds to, respectively, 38–43 per cent of the abovementioned total extracellular Na^+^ pool (31.5 *μ*moles (g wet wt.)^−1^ available for excitation-induced uptake into the muscle cells.

**Figure 7:**
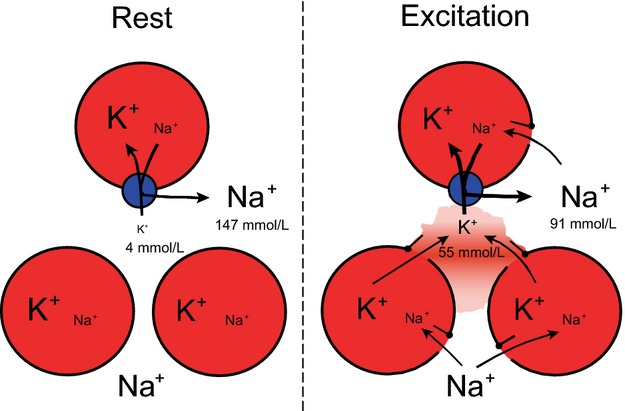
Diagram of Na^+^, K^+^ distribution in rat EDL muscle at rest and during electrical stimulation at 60 Hz for 60 sec. The stimulation leads to opening of the Na^+^-channels allowing rapid entry of Na^+^ ions into the muscle cells, causing depolarization, and rapid efflux of K^+^ ions via voltage-sensitive K^+^ channels. The implication of this redistribution is that in the interstitial water space Na^+^ decreases from 147 to 91 mmol L^−1^ and K^+^ increases from 4 to 55 mmol L^−1^, sufficient to cause rapid decrease in excitability and muscle fatigue, which is restored primarily by acceleration of active electrogenic Na^+^, K^+^ transport via the Na^+^, K^+^ pump, indicated in blue (Clausen 2003).

As summarized in Figure7, 60 sec of 60 Hz stimulation induces an intracellular uptake of 12 *μ*moles (g wet wt.)^−1^ of ^22^Na leading to a rapid drop in the content of Na^+^ in the extracellular pool of 12 *μ*moles (g wet wt)^−1^/0.214 mL = 56 *μ*moles (mL)^−1^ = 56 mmol L^−1^, reaching an average Na^+^ concentration of 91 mmol L^−1^ (147–56 mmol L^−1^) in the interstitial water space between the cells. This drop is replaced by an almost equimolar amount of K^+^ (11.0 *μ*moles (g muscle wet wt.)^−1^, Fig.3A), increasing [K^+^]_o_ by 51 mmol L^−1^ (11 *μ*mol (g wet wt.)^−1^/0.214 mL (Fig.3B) reaching 4 + 51 = 55 mmol L^−1^, similar to the drop in [Na^+^]_o_ of 56 mmol L^−1^.

As shown in Figure2A, after 60 sec of 60 Hz stimulation (3600 pulses), ^22^Na uptake reaches 19.5 *μ*mol/g wet wt. (5.4 nmol (g wet wt.)^−1^/pulse). This is in good agreement with our previously measured rate of ^22^Na uptake in rat EDL muscle stimulated at 40 Hz (5.8 nmol (g wet wt.)^−1^/pulse, (Gissel and Clausen 2000). In this study (Fig.2B), the intracellular Na^+^ content (22.8 *μ*moles (g wet wt.)^−1^ reached after 60 sec of 60 Hz stimulation corresponds to an intracellular Na^+^ concentration of 42 mmol L^−1^ (22.8 *μ*moles/0.544 mL). This, combined with the increase in [K^+^]_o_ of 51 mmol L^−1^ (Fig.3B), is likely to induce a marked increase in Na^+^, K^+^ pumping rate. As already stated, the extracellular space of the EDL muscle contains a reservoir of Na^+^ (31.5 *μ*moles (g wet wt.)^−1^ available for the generation of action potentials for a limited period. 38–43% of that Na^+^ reservoir can be utilized to maintain excitation for 60 sec of stimulation at 60 Hz. Previous experiments showed that 60 sec of 60 Hz stimulation decreased total K^+^ content of EDL muscle by 11 *μ*mol (g wet wt.)^−1^ and augmented Na^+^ content by 11.2 *μ*mol (g wet wt.)^−1^. These changes were completely restored during 10 min of subsequent rest (Clausen 2013; Table2 and Fig.1). As described below, long before this complete restoration, substantial net efflux of Na^+^ was observed. Thus, earlier studies showed that excitation of rat EDL for 5 sec at 90 Hz caused a net loss of K^+^ of 11.4 nmol (pulse)^−1^ (Clausen et al. 2004). Hence, it would be expected that 15 sec of stimulation at 60 Hz causes a net loss of K^+^ of 11.4 × 15 × 60 = 10,260 nmol (g wet wt.)^−1^ = 10.3 *μ*mol (g wet wt.)^−1^, in good agreement with the net loss of K^+^ of 9.4 *μ*mol (g wet wt.)^−1^ observed after 15 sec in the present study (Fig.3A). As shown in Figure3A, the subsequent (15–60 sec) net loss of K^+^ is much lower. As discussed in the introduction this is likely to reflect the inhibitory effect of progressive rise in [K^+^]_o_. Also in vivo 60 Hz of stimulation caused a rapid early (0–15 sec) rise in net loss of K^+^ (Fig.6A) and [K^+^]_o_ (Fig.6B), followed by a plateau with no further increase.

The present observations of large excitation-induced passive fluxes of Na^+^ and K^+^ indicate that during work, excitability may primarily be maintained by rapid and marked stimulation of the electrogenic Na^+^, K^+^ pumps, allowing repolarization. Experiments with isolated rat soleus muscles showed that stimulation at 60 Hz for 10 sec increases intracellular Na^+^ content by 58% (Everts and Clausen 1994). Within the following 110 sec after the cessation of stimulation, net extrusion of Na^+^ amounted to 4429 nmol (g wet wt.)^−1^ (min)^−1^, corresponding to 47% of the maximum Na^+^, K^+^ pumping rate at 30°C. This confirms the rapid net decrease in intracellular amount of Na^+^ (3500 nmol/g wet wt./min) measured using Na^+^-sensitive microelectrodes in mouse soleus after intense electrical stimulation (Juel 1986). In isolated rat soleus it was observed that after 10 sec of stimulation at 120 Hz, the net Na^+^ re-extrusion measured in the subsequent 30 sec of rest reaches a 22-fold increase in Na^+^, K^+^ pump activity corresponding to 97% of the theoretical maximum rate of active Na^+^, K^+^ pumping measured and calculated on the basis of total content of ^3^H-ouabain binding sites (Nielsen and Clausen 1997). In rat EDL muscles 15 sec of 60 Hz stimulation increased total cellular Na^+^ content to 18.0 *μ*mol (g wet wt.)^−1^. In the first 60 sec after this stimulation, Na^+^ content decreased to 14.6 *μ*mol/g wet wt., corresponding to a net Na^+^ extrusion of 3400 nmol (g wet wt)^−1^ (min)^−1^. This flux is 7.3-fold larger than the ^22^Na^+^ efflux determined in resting rat EDL muscles (Clausen 2011). These examples show that even isolated muscles during or after electrical stimulation rapidly reach very high rates of active Na^+^, K^+^-transport, indicating that excitation is one of the most potent stimuli for the Na^+^,K^+^ pumps. Thus, the population of Na^+^, K^+^ pumps in the muscles may promptly approach considerable functionality allowing adequate compensation of the large excitation-induced passive Na^+^, K^+^-fluxes demonstrated over the recent decades. A later study (Clausen 2013) showed that in rat EDL muscle in vivo stimulation for 300 sec at 5 Hz caused a net loss of K^+^ of 12.6 *μ*mol/g wet wt, sufficient to increase [K^+^]_o_ by 12.6 *μ*mol/0.177 mL = 71 mmol L^−1^ (*P* < 0.007), indicating that even at a lower and more physiological frequency, [K^+^]_o_ may reach a level sufficient to cause complete loss of excitability, albeit five times slower (300/60) than during 60 Hz stimulation for 60 s.

K^+^-induced inhibition of excitability seems to take place predominantly in the t-tubules of the muscle (Lindinger 2005). In rat EDL the volume of the t-tubular lumen amounts to 1.41% of total muscle volume (Launikonis and Stephenson 2002). This implies that the tubular system in 1 g of muscle contains 14.1 *μ*L of water. In rat EDL muscle, each action potential causes a net loss of 11.4 nmol of K^+^ (g wet wt.)^−1^ into the surrounding water space (Clausen et al. 2004). This amount of K^+^ is primarily released from the cytoplasm into the t-tubular volume, increasing the average luminal concentration of K^+^ by 11.4 nmol/14.1 *μ*L = 0.81 nmol/*μ*L = 0.81 *μ*mol/mL, corresponding to 0.81 mmol L^−1^. During stimulation at 60 Hz, this would correspond to 60 × 0.81 mmol L^−1^ = 48.6 mmol L^−1^/sec elevating luminal K^+^ from 4 to 48.6 mmol L^−1^ in one sec, more than enough to block excitation. However, before luminal K^+^ reaches such a high value, excitability is likely to be lost. As contractions usually continue appreciably longer, rapid mechanisms seem to maintain excitability. First and most importantly, the increase in intracellular Na^+^ causes prompt and often marked activation of the electrogenic Na^+^, K^+^ pumps (Everts and Clausen 1994; Nielsen and Clausen 1997), with repolarization favoring re-accumulation of K^+^ into the cytoplasm. Second, the initial depolarization favors the influx of Cl^−^, leading to partial repolarization and increased uptake of K^+^ (Hodgkin and Horowicz 1959). Third, excitability is improved by the excitation-induced reduction in G_Cl_ (Pedersen et al. 2005), mediated by activation of protein kinase C (Pedersen et al. 2009). The present results support and extend previous evidence that in rat EDL muscle, excitation induces up to 45–70 mmol L^−1^ increase in [K^+^]_o_, sufficient to cause fatigue by depolarization interfering with further excitation. This might explain why muscles have developed their surprisingly high capacity for active Na^+^, K^+^ transport allowing (depending on the frequency of excitation) partial or complete clearance of the excitation-induced rise in extracellular K^+^ and force recovery within seconds (Clausen et al. 1987; Everts and Clausen 1994; Nielsen and Clausen 1997).

## Conclusions

When taken together, the present observations indicate that excitation induces an early influx of Na^+^, quantified using ^22^Na as well as by flame photometry (Fig.2A and B). This is associated with a similar efflux of K^+^, leading to progressive accumulation of K^+^ in the extracellular space (Fig.3B). Both [Na^+^]_i_ and [K^+^]_o_ reach a plateau with modest further changes, indicating that the Na^+^, K^+^ fluxes are self-limiting, probably reflecting depolarization and ensuing weakening or cessation of action potentials. These phenomena are due to the excitation-induced drop in [Na^+^]_o_ (here estimated to 56 mmol L^−1^) as well as the increase in [K^+^]_o_ of 55 mmol L^−1^ acting synergistically to develop fatigue. This is the cause of choosing the expression “sodium–potassium fatigue” for the running title of this manuscript, also because this new term identifies the general physiological significance and the translational perspective of this study.
